# CRISPR/Cas9 knockout of MTA1 enhanced RANKL‐induced osteoclastogenesis in RAW264.7 cells partly via increasing ROS activities

**DOI:** 10.1111/jcmm.17692

**Published:** 2023-02-14

**Authors:** Mingzhe Feng, Lin Liu, Zechao Qu, Bo Zhang, Yanjun Wang, Liang Yan, Lingbo Kong

**Affiliations:** ^1^ Department of Spine Surgery Xi'an Honghui Hospital, School of Medicine, Xi'an Jiaotong University Xi'an China; ^2^ Department of Critical Care Medicine Xi'an Honghui Hospital, School of Medicine, Xi'an Jiao Tong University Xi'an China; ^3^ Department of Emergency Xi'an Honghui Hospital, School of Medicine, Xi'an Jiaotong University Xi'an China

**Keywords:** CRISPR/Cas9, metastasis‐associated protein 1, osteoclastogenesis, RAW264.7

## Abstract

Metastasis‐associated protein 1 (MTA1), belonging to metastasis‐associated proteins (MTA) family, which are integral parts of nucleosome remodelling and histone deacetylation (NuRD) complexes. However, the effect of MTA1 on osteoclastogenesis is unknown. Currently, the regulation of MTA1 in osteoclastogenesis was reported for the first time. MTA1 knockout cells (KO) were established by CRISPR/Cas9 genome editing. RAW264.7 cells with WT and KO group were stimulated independently by RANKL to differentiate into mature osteoclasts. Further, western blotting and quantitative qRT‐PCR were used to explore the effect of MTA1 on the expression of osteoclast‐associated genes (including CTSK, MMP9, c‐Fos and NFATc1) during osteoclastogenesis. Moreover, the effects of MTA1 on the expression of reactive oxygen species (ROS) in osteoclastogenesis was determined by 2′, 7′ ‐dichlorodihydrofluorescein diacetate (DCFH‐DA) staining. Nuclear translocation of Nrf2 was assessed by immunofluorescence staining and western blotting. Our results indicated that the MTA1 deletion group could differentiate into osteoclasts with larger volume and more TRAP positive. In addition, compared with WT group, KO group cells generated more actin rings. Mechanistically, the loss of MTA1 increased the expression of osteoclast‐specific markers, including c‐Fos, NFATc1, CTSK and MMP‐9. Furthermore, the results of qRT‐PCR and western blotting showed that MTA1 deficiency reduced basal Nrf2 expression and inhibited Nrf2‐mediated expression of related antioxidant enzymes. Immunofluorescence staining demonstrated that MTA1 deficiency inhibited Nrf2 nuclear translocation. Taken together, the above increased basal and RANKL‐induced intracellular ROS levels, leading to enhanced osteoclast formation.

## 1 INTRODUCTION

Bone is a kind of dynamic yet stable connective tissue that remodels constantly throughout life, which is influenced by the activities of two main cell populations, osteoclast‐mediated bone resorption and osteoblast‐mediated bone formation.^1^ Absorption and formation are stable under physiological conditions. However, when the balance between bone formation and resorption is disturbed, which will lead to a disorder of bone homeostasis. Osteoblasts (OBs) are mainly derived from mesenchymal progenitor cells in the inner and outer periosteum and bone marrow matrix, which can specifically secrete a variety of bioactive substances to regulate and influence the process of bone formation and reconstruction.^2^ Osteoclasts (OCs) originate from haematopoietic stem cells (HSCs), are a type of multinucleated giant cells (MNCs) that specializes in absorbing organic collagen and mineralized matrix.^3^ Excess osteoclastic activity is a major cause of bone skeletal metabolic diseases, such as osteoporosis (OP), periodontitis, rheumatoid arthritis and other osteolysis‐related diseases. Clinical anti‐osteolysis drugs such as bisphosphonates, oestrogens, serotonin and calcitonin, but long‐term treatment with these drugs may cause many restrictions and side effects such as impaired fracture healing, increased risk of breast cancer, osteonecrosis of the jaw, the risk of venous thromboembolism and neurological reactions.^4^, ^5^ In addition, most US Food and Drug Administration (FDA)‐approved drugs treat osteolysis by inhibiting osteoclasts are still limited.^6^, ^7^ Therefore, it is important to elucidate intricate molecular mechanisms that regulate osteoclast differentiation and function to improve the treatment of the pathogenesis of bone diseases.

OCs are regulated by multiple hormones and local cytokines. However, receptor activator of nuclear factor‐κB ligand (RANKL) and macrophage colony stimulating factor (M‐CSF) are the key molecules of OCs differentiation.^8^ The RANKL/RANK interaction with M‐CSF/c‐Fms triggers the activation of multiple downstream signalling pathways that are essential for osteoclast formation, such as nuclear factor κB (NF‐κB) signalling, mitogen‐activated protein kinase (MAPK) signalling, PI3K‐protein kinase B (AKT) signalling, Ca^2+^‐calcineurin‐NFATc1 signalling and reactive oxygen species (ROS) signalling, etc.^6^ Further activating the master transcription factors c‐Fos and nuclear factor of activated T‐cells 1 (NFATc1), which can induce the expression of osteoclast‐specific genes including tartrate‐resistant acid phosphatase (TRAP), cathepsin K (CTSK), matrix metallopeptidase‐9 (MMP‐9) and osteoclast stimulatory transmembrane protein (OC‐STAMP) contributes to osteoclast activation and maturation.^9^ Normal metabolism of the body can produce reactive oxygen species, containing superoxide anion radical (O_2_^·−^), hydrogen peroxide (H_2_O_2_), hydroxyl radical (−OH) and nitric oxide (NO).^10^ In addition, recent studies have shown that ROS are important components in RANKL‐mediated osteoclast differentiation and maturation.^11^

Metastasis‐associated protein 1 (MTA1), belonging to metastasis‐associated proteins (MTA) family, which are integral parts of nucleosome remodelling and histone deacetylation (NuRD) complexes.^12^ MTA1 is mainly studied in cancer, and it plays a role in the transformation, invasion, survival, DNA repair, angiogenesis, hormone dependence and treatment resistance of cancer.^13^ Besides strong correlation between MTA1 upregulation and cancer, growing evidence strongly suggests that MTA1 could regulate divergent cell pathways by modifying status of crucial target genes under both pathological and physiological statuses.^14^ It has been confirmed that MTA1 is involved in normal physiological processes such as embryonic development, spermatogenesis during reproduction, cell ageing, nervous system and photosensory system.^15^ It has been reported that MTA1 can stimulate the growth of osteoblasts under hypoxia, but inhibit the differentiation of MC3T3 cells, which may promote the accumulation of osteoblasts in bone remodelling.^16^ However, physiological characteristics of MTA1 have still been poorly addressed, especially the effects of MTA1 on osteoclast differentiation has not been reported so far.

CRISPR/Cas 9 is a method for selectively editing target genes by Cas 9 proteins directed by RNA at the genomic level. Due to the simple operation and less time, applications are becoming more and more widespread. It plays an important role in gene knockdown, regulated expression levels of genes and gene editing therapy for diseases.^17^ In this study, we constructed RAW264.7 macrophage line with MTA1 gene knockout by technology to investigate the function of MTA1 in osteoclasts, and it is of great significance for the treatment of bone diseases to further understand its mechanism in osteoclast differentiation and bone catabolism. Our results indicate that MTA1 plays a pivotal role in RANKL‐induced osteoclast differentiation and negatively regulates osteoclast differentiation via affecting ROS production and antioxidant production. It also provides a potential molecular target for the treatment of osteoclast‐mediated bone metabolic diseases.

## 2 MATERIALS AND METHODS

### 2.1 Cell culture and osteoclast differentiation

Bone marrow macrophages (BMMs)/Murine macrophage cell line RAW264.7 cells were purchased from American Type Culture Collection (ATCC, Manassas, VA). BMMs and RAW264.7 cells were cultured in DMEM (Hyclone, Logan, UT, USA) containing 10% FBS (Gibco, Rockville, MD, USA) in humidified air with 5% CO_2_ at 37°C. To induce osteoclasts, as described in our previous study,^18^ BMMs were cultured for 5 days after adding M‐CSF (20 ng/mL) and RANKL (40 ng/mL) to the culture system. RAW264.7 cells were cultured in α‐minimal essential medium (α‐MEM, Hyclone) with RANKL (40 ng/mL) for 5 days.

### 2.2 Tartrate‐resistant acid phosphatase (TRAP) staining

The culture supernatants were inoculated into the 96‐well plate. To identify osteoclasts, the cells were fixed with 4% paraformaldehyde for 15 min, washed with phosphate‐buffered saline (PBS) three times and stained with TRAP kit (Sigma‐Aldrich, USA) according to the manufacturer's protocol for 60 min at 37°C in the dark. The TRAP‐positive multinucleated cells (≥3) were visualized and counted under light microscopy (Leica, Germany). The number of TRAP‐positive cells were counted and records for each well.

### 2.3 Cell proliferation assay

RAW264.7 cells were cultured at a density of 3 × 10^3^ cells per well into 96‐well plates and stimulated with 40 ng/mL RANKL. At 0, 24, 48 and 72 h of cell culture, 10 μL Cell Counting Kit‐8 (CCK‐8) solution (Beyotime, Shanghai, China) was added and incubated for 3 h to study the proliferation of the RAW264.7 cells. Then, the optical density (OD) at 450 nm was measured using a 96‐well microplate reader (Multiskan FC; Thermo Scientific, USA).

### 2.4 Flow cytometric analysis

The effect of MTA1 knockout on the apoptosis of RAW264.7 cells was detected by flow cytometry. Annexin V‐FITC/PI Apoptosis Detection Kit was used following the protocol provided by the manufacturer (C1062L, Beyotime, China). RAW264.7 cells were seeded in 6‐well plates and grown for 48 h to a suitable density, digested with 0.25% EDTA‐free trypsin, washed three times with precooled PBS and collected by centrifugation at 200 r/min. Cells were then washed twice with PBS, centrifuged twice at 200 r/min and the supernatant was discarded. The cells were resuspended by adding 195 μL Annexin V‐FITC binding solution, followed by adding 5 μL Annexin V‐FITC and 10 μL propidium iodide staining solution, mixed gently and then incubated at room temperature in the dark for 20 min. Finally, the cells were analysed by flow cytometry (Becton‐Dickenson, USA).

### 2.5 F‐actin ring immunofluorescence

RAW264.7 cells were cultured in 96‐well plates and performed cytoskeletal fibrous actin (F‐actin) staining to observe sealing zone formation. After induction, the cells were fixed with 4% paraformaldehyde for 15 min, washed by PBS three times then permeabilized with 0.1% Triton X‐100 for 10 min and stained with Rhodamine‐phalloidin (Yeasen, Shanghai, China) in the dark for 40 min at room temperature. The nuclei were then stained and incubated with 4′,6‐diamidino‐2‐phenylindole (DAPI) for 10 min. Sealing zone formation and nuclear number were visualized by fluorescence microscope (Leica, Germany).

### 2.6 Measurement of intracellular ROS production

ROS assay kit (Beyotime) was used to detect the intracellular production of ROS based on the cell permeant fluorogenic dye, 2,7‐dichlorodihydrofluorescein diacetate (DCFH‐DA). Briefly, the RAW264.7 cells were seeded into a 96‐black well plate for 24 h and then pre‐treated with 40 ng/mL RANKL. Cells were incubated with 10 μM DCFH‐DA in the dark for 30 min at 37°C, rinsed in culture media three times and the fluorescence of DCF was measured using a fluorescence microscope (Leica). And the mean fluorescence intensity was quantitatively analysed by ImageJ software.

### 2.7 Immunofluorescence (IF) staining assay

RAW264.7 cells were cultured in chamber slides at a density of 5 × 10 cells per well for 12 h and then treated with 40 ng/mL RANKL for 24 h. For immunofluorescence staining, the cells were incubated with anti‐Nrf2 (1:200) for 2 h, followed by FITC‐labelled goat anti‐rabbit IgG (H + L) antibody (Beyotime) for 30 min. Finally, the cells were stained with DAPI solution for 10 min and then observed, and images were acquired under a confocal microscope (Nikon, Japan).

### 2.8 Western blotting analysis

Total proteins from BMMs or RAW264.7 cells were isolated with RIPA buffer (Beyotime) containing PMSF and phosphatase inhibitors centrifuged at 13,000 × g for 5 min. The lysates were collected, and the protein concentration was quantitatively analysed using BCA assay kit (Mishu, Xi'an, China). Cellular proteins were resolved by sodium dodecyl sulphate‐polyacrylamide gel electrophoresis (SDS‐PAGE). Then the proteins were transferred to polyvinylidene difluoride (PVDF) membranes (Merck Millipore, Billerica, MA, USA). The membranes were blocked with 5% non‐fat milk for 1 h, and then probed at 4°C overnight with primary antibodies against c‐Fos, NFATc1 (Abcam, Cambridge, MA), MTA1(Affinity, Jiangsu, China), NRF2, HO‐1, CAT, GCLC (Cell Signalling Technology, Beverly, MA, USA), CTSK and MMP9 (Beyotime). Subsequently, membranes were incubated with horseradish peroxidase‐conjugated secondary antibody (Beyotime) for 2 h. Finally, the signals of target proteins were visualized using the enhanced chemiluminescence reagent (Beyotime) and the band intensity was analysed with ImageJ software. Band intensity was normalized to the corresponding β‐tubulin (Beyotime).

### 2.9 Real‐time quantitative polymerase chain reaction (RT‐qPCR)

RAW264.7 cells were inoculated into 6‐well plates and stimulated with RANKL (40 ng/mL) for a period. BMMs were seeded in 6‐well plates and stimulated with M‐CSF (20 ng/mL) and RANKL (40 ng/mL) for 5 days. Then, total RNA was extracted by TRIzol method according to the manufacturer's instructions. Prime Script RT kit was then used for reverse transcription into cDNA. The obtained cDNA was used as a template for real‐time quantitative PCR on the Light Cycler® 96 system (Roche, Basel, Switzerland). The primers employed in this study are listed in Table 1. The housekeeping gene GAPDH was used as internal control.

**TABLE 1:** Primer sequences used for qRT‐PCR analysis.

Target genes	Primer sequence (5′‐3′) forward	Primer sequence (5′‐3′) reverse
c‐Fos	TGTTCCTGGCAATAGCGTGT	TCAGACCACCTCGACAATGC
NFATc1	TCAGAGTGAGACCGAGAGGC	TGACATGCGGGGTGTGTG
MMP9	GTTAGCCAGAAGCTGCGGT	GGGGAAGACCACAAAAGTCG
Cathepsin K	CTGCGGCATTACCAACATGG	ACTGGAAGCACCAACGAGAG
MTA1	CTGAGATCCAGGCCCAAGTG	TGGACTGTGACCCATTTCCG
Nrf2	GGCTCAGCACCTTGTATCTT	CACATTGCCATCTCTGGTTTG
CAT	CAGATGGAGAGGCAGTCTATTG	AAAGATCTCGGAGGCCATAATC
GCLC	TTTAAGCCTCCTCCTCCAAAC	CTAGTGAGCAGTACCACGAATAC
HO‐1	CAGCATCCCAAAGTCAATCAAG	GCATAAACTCCCATTCCAACAG

### 2.10 CRISPR/Cas9‐mediated MTA1 knockout

Deletion of MTA1 in RAW264.7 cell lines was accomplished by Cyagen Biosciences Inc. US (China‐branch). In brief, the MTA1 gene sequence was obtained from NCBI database, and the appropriate gRNA oligonucleotide was designed and constructed. Cas9 protein and gRNA oligonucleotide were transferred into RAW264.7 cell line via electroporation using CRISPR/Cas9 gene editing technique. CRISPR/Cas9‐mediated MTA1 deletion monoclonal cells were obtained by PCR amplification. Finally, Sanger sequencing was used for homozygous verification.

### 2.11 Statistical analysis

Data are expressed as mean ± standard deviation (SD). Differences between two groups were evaluated using Student's *t*‐test, and multiple group comparisons were tested using one‐way anova, statistical analyses were performed using the GraphPad Prism software, *p < 0.05 was considered as a statistically significant difference.

## 3 RESULTS

### 3.1 MTA1 expression were decreased during osteoclast differentiation

**FIGURE 1:**
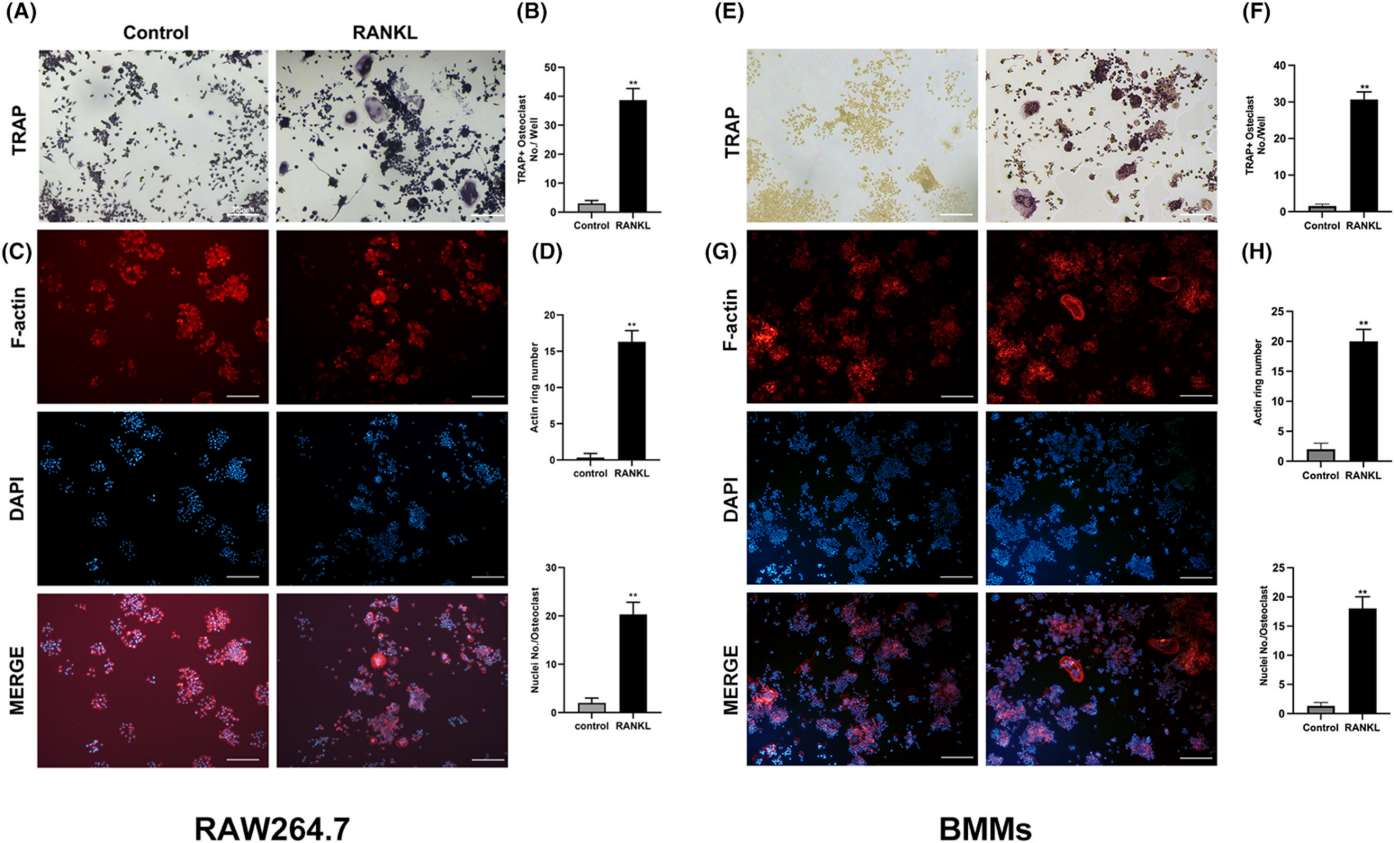
RANKL independently induced the differentiation and formation of multinucleated osteoclasts. (A) RANKL‐stimulated RAW264.7 cells were stained with TRAP, and representative images are shown (Scale bar = 300 μm). (B) Mature osteoclasts were quantified by the number of TRAP^+^ multinucleated (nuclei ≥3) cells in each group. (C) RANKL independently stimulated osteoclast differentiation, and fluorescent images were stained with Phalloidin‐FITC (F‐actin) and DAPI (nuclei). (Scale bar = 300 μm). (D) Quantification analyses of F‐actin ring and nuclei number per osteoclast. (E) M‐CSF and RANKL‐stimulated BMMs were stained with TRAP, and representative images are shown (Scale bar = 300 μm). (F) TRAP+ multinucleated (≥3 nuclei) cell count was used to detect the number of mature osteoclasts induced by BMMs. (G) M‐CSF and RANKL stimulated BMMs to differentiate into osteoclasts, and fluorescent images were stained with Phalloidin‐FITC (F‐actin) and DAPI (nucleus). (Scale bar = 300 μm). (H) Quantification analyses of F‐actin ring and nuclei number per osteoclast. Data are expressed as mean ± SD; *p < 0.05 and **p < 0.01 versus control group.

**FIGURE 2:**
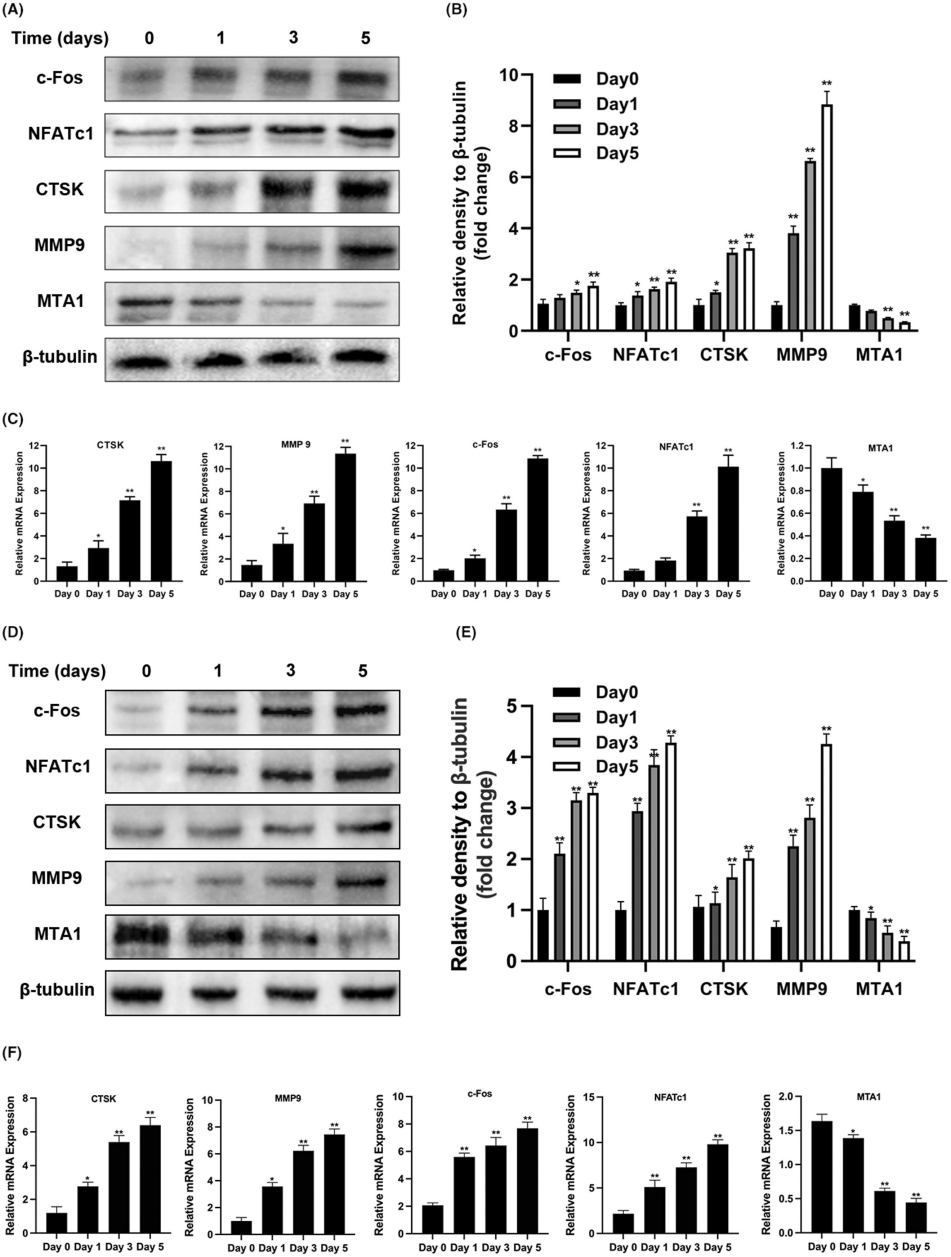
Expression of MTA1 in RAW264.7 cells and BMMs during osteoclastogenesis induced by RANKL. RAW264.7 cells were independently stimulated with RANKL (40 ng/mL), and then total protein or RNA was extracted according to incubation times (days 0, 1, 3 and 5); BMMs were stimulated with M‐CSF (20 ng/mL) and RANKL (40 ng/mL) and cultured for 5 days. Total protein or RNA was extracted according to the culture time (days 0, 1, 3 and 5). (A, B) Western blot and ImageJ analysis were used to detect the protein expression of c‐Fos, NFATc1, CTSK, MMP9 and MTA1 in RAW264.7 cells. (C) qRT‐PCR was used to detect the mRNA expression levels of c‐Fos, NFATc1, CTSK, MMP9 and MTA1 during RAW264.7 differentiation. (D, E) Western blot and ImageJ analysis were used to detect the protein expression of c‐Fos, NFATc1, CTSK, MMP9 and MTA1 in BMMs. (F) qRT‐PCR was used to detect the mRNA expression levels of c‐Fos, NFATc1, CTSK, MMP9 and MTA1 during BMMs differentiation. Data are expressed as the means ± SD of three independent experiments; *p < 0.05, **p < 0.01 versus control group.

Although many document studies have been reported that the differentiation of RAW264.7 cells to multinucleated osteoclastic cells requires M‐CSF and RANKL co‐stimulation, Song et al. (2018) reported that after seeded RAW264.7 with the density of 6.25 × 10^3^ cells/cm^2^ and treatment with RANKL independently at 12 h, the number of multinucleated osteoclast cells were significantly increased.^8^ Based on this, RANKL was used to independently induce the differentiation of RAW264.7 into osteoclasts. However, to induce osteoclast differentiation of BMMs, both M‐CSF and RANKL were added to the culture medium. Our staining analysis showed that after RANKL independently stimulated RAW264.7 cells for 5 days, the number of TRAP‐positive osteoclasts and the number of actin rings in mature osteoclasts gradually increased (Figure 1A–D). Similar results were observed in BMMS‐induced osteoclasts (Figure 1E–H). Furthermore, the expression of osteoclast‐specific genes and proteins such as CTSK, MMP9, c‐Fos and NFATc1 increased significantly with cell differentiation. However, at the protein and molecular levels, the expression of MTA1 protein and gene decreased gradually with the progress of osteoclast differentiation and maturation in a time‐dependent manner. The above results were generally consistent in the process of osteoclast differentiation induced by BMMs (Figure 2A–C) and RAW264.7 (Figure 2D–F). These results indicate that MTA1 may be involved in the negative regulation of osteoclastogenesis.

### 3.2 Construction of stable MTA1 knockout RAW264.7 cell line using CRISPR/Cas9 programme

**FIGURE 3:**
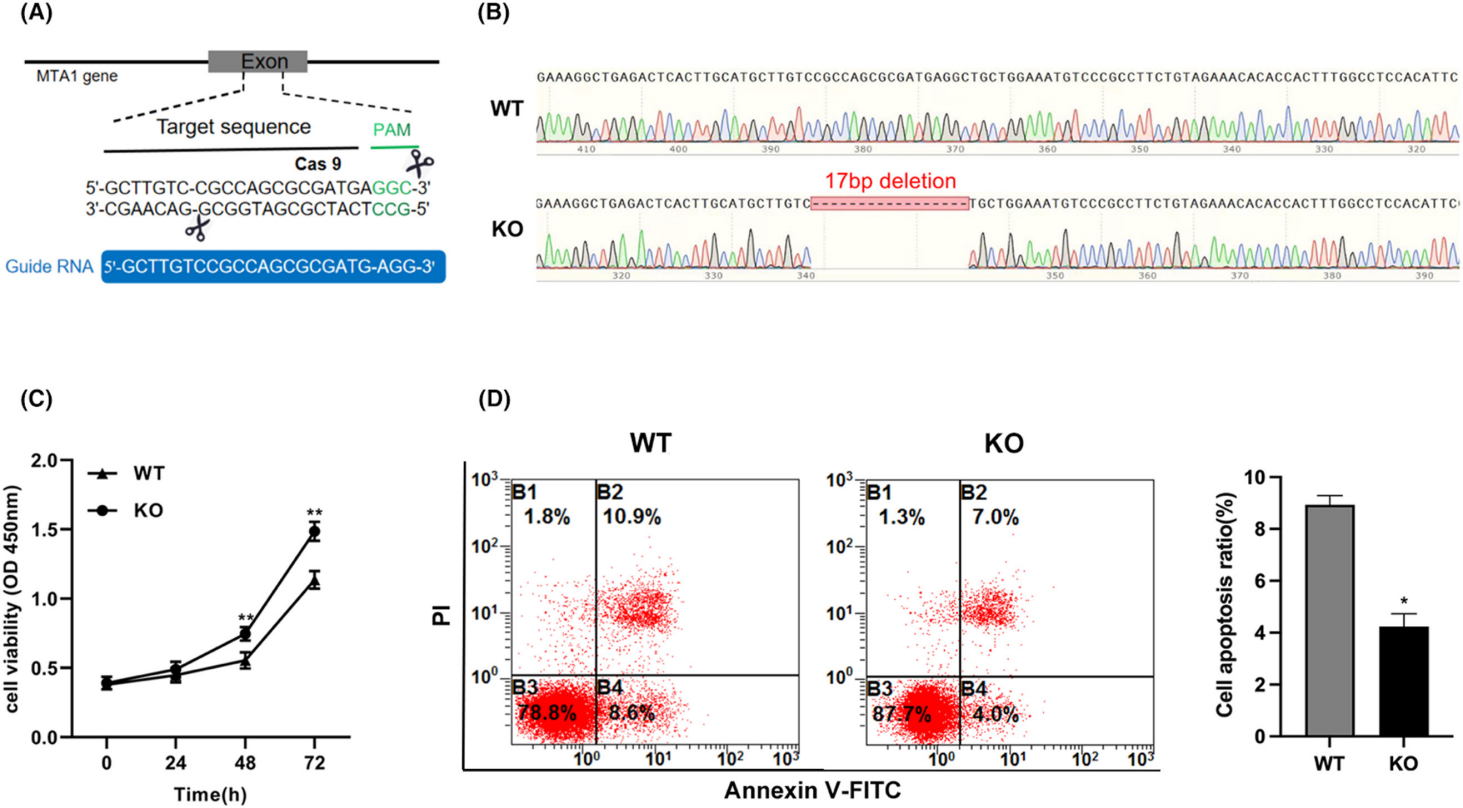
The establishment of MTA1 gene knockout in RAW264.7 cells via CRISPR/Cas9 gene editing technique. (A) Schematic diagram of targeting strategy for inactivated MTA1 gene. The gRNA sequence (blue) pairs with the DNA target (black), directly upstream of the adjacent motif (PAM sequence, green). (B) Sanger sequencing proved the presence of the genetic modification in the heterogeneous population of cells transduced with electroporation containing CRISPR/Cas9 gRNA against MTA1. (C) The growth curve of RAW264.7 cells in WT group and KO group was determined by CCK‐8 method. (D) The apoptosis of RAW264.7 after MTA1 knockdown was assessed by flow cytometric analysis. Data are expressed as the means ± SD of at least three independent experiments; *p < 0.05, **p < 0.01 versus WT group.

In order to further determine the specific regulatory role of MTA1 in osteoclast differentiation, we establish MTA1 knockout cells (KO). KO cells were established in mouse macrophage lines RAW264.7 without RANKL treatment using the CRISPR/Cas9 gene editing technique. Sequencing results verified that mouse MTA1 gene knockout homozygous cells were successfully established (Figure 3A, B). First, to investigate the effect of MTA1 knockout on RAW264.7 cells, we assessed cell viability at 0, 24, 48 and 72 h after RANKL stimulation. The results of CCK‐8 showed that MTA1 knockout promoted the differentiation of RAW264.7 cells, especially at 48 and 72 h of cell culture (Figure 3C). Subsequently, to explore the impact of MTA1 knockout on RAW264.7 apoptosis, flow cytometric analysis was conducted. The results suggested that MTA1 knockout reduced the RAW264.7 apoptosis rate (Figure 3D).

### 3.3 Deficiency of MTA1 accelerated osteoclast differentiation

**FIGURE 4:**
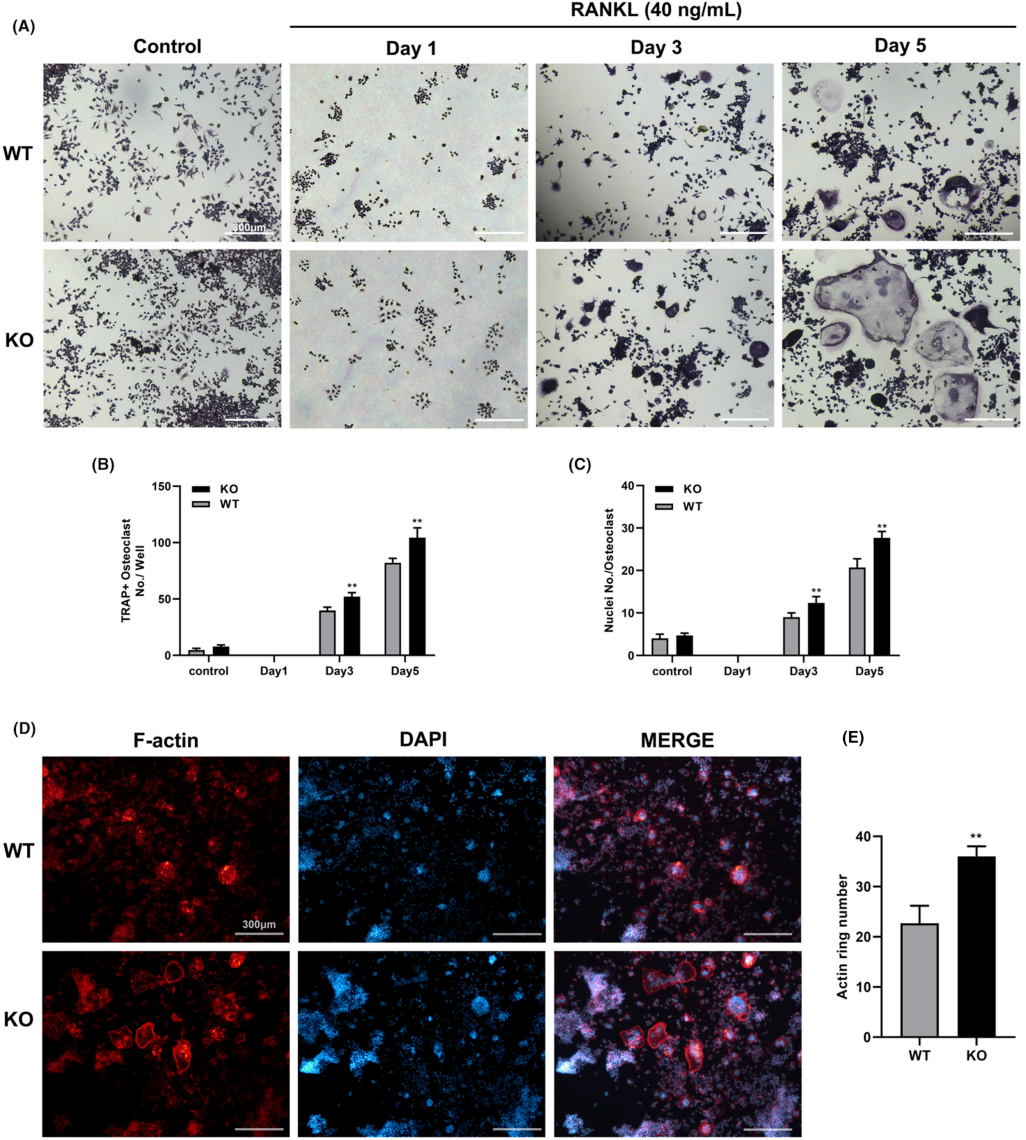
MTA1 deficiency improved osteoclast differentiation and F‐actin ring formation. (A) RAW264.7 cells with WT and KO groups were independently induced with RNAKL (40 ng/mL) for 5 days. Representative TRAP‐positive mature osteoclasts are shown (Scale bar = 300 μm). (B) Number of TRAP‐positive osteoclasts per well. (C) The number of nuclei per osteoclast. (D) RAW264.7 cells with WT and KO groups were incubated RANKL for 5 days. Representative Phalloidin‐FITC fluorescent staining images are shown (Scale bar = 300 μm). (E) Quantification analyses of actin ring number. Data are expressed as the means ± SD of at least three independent experiments; *p < 0.05, **p < 0.01 versus WT group.

After osteoclast differentiation in vitro was analysed using RAW264.7 KO and WT groups to evaluate the role of MTA1 in RANKL‐induced osteoclast differentiation. Interestingly, the results were in line with our expectations. Morphologically, there were significant differences between the two groups in cell size and nucleus numbers of fused cells. Compared with the WT group, the MTA1 KO group formed more TRAP‐positive multinucleated osteoclasts and the number and size of osteoclasts formed were larger with the induction of osteoclast differentiation time (Figure 4A–C). Furthermore, the bone resorption function of mature osteoclasts depends on the reorganization of the actin cytoskeleton, which then forms sealing zone and ruffled border that binds osteoclasts tightly to the bone surface. Sealing zone is one of the critical functional domains of osteoclast bone absorption; therefore, we performed F‐actin assay to evaluate the effect of MTA1 on the formation of the sealing zone structure. Consistent with the results of TRAP staining, F‐actin staining showed that the number of actin rings formed in the MTA1 KO group increased after RANKL treatment compared to the WT group (Figure 4D, E).

### 3.4 Deletion of MTA1 promoted the expression of osteoclast‐specific genes

**FIGURE 5:**
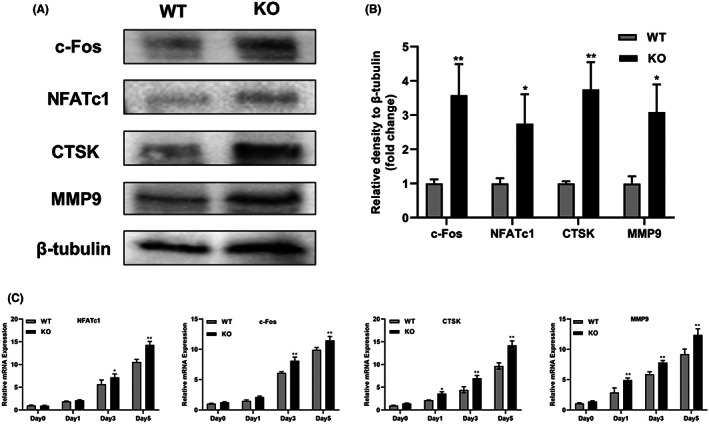
Deletion of MTA1 promoted the expression of osteoclast‐specific gene. (A, B) c‐Fos, NFATc1, CTSK and MMP9 protein expression RAW264.7 cells with WT and KO groups during osteoclast differentiation at day 3 were detected and quantified by western blot and ImageJ analysis. (C) mRNA expression of NFATc1, c‐Fos, CTSK and MMP9 in RAW264.7 cells with WT and KO groups treated with RANKL (40 ng/mL) for the indicated times (days 0, 1, 3 and 5) were quantitatively measured by qRT‐PCR. Data show means ± SD of at least three independent experiments; *p < 0.05, **p < 0.01 versus WT group.

To further compare the expression levels of osteoclast differentiation‐related genes in KO group and WT group, we used western blotting and PCR to analyse the protein and mRNA expression levels of osteoclast differentiation‐related genes. Total RNA was extracted at 0, 1, 3 and 5 days after RANKL induction, as shown in Figure 5C, the expression levels of osteoclast‐associated genes (including CTSK, MMP9, c‐Fos and NFATc1) were significantly increased in KO group. The difference between the two groups was more pronounced on day 3 and 5. In addition, compared with WT group, the protein levels of CTSK, MMP9, c‐Fos and NFATc1 were significantly increased on day 3 in KO cells (Figure 5A, B). These results suggested that MTA1 deficiency promoted osteoclast differentiation and functional expression.

### 3.5 Lack of MTA1 facilitated RANKL‐induced ROS production in osteoclastogenesis

**FIGURE 6:**
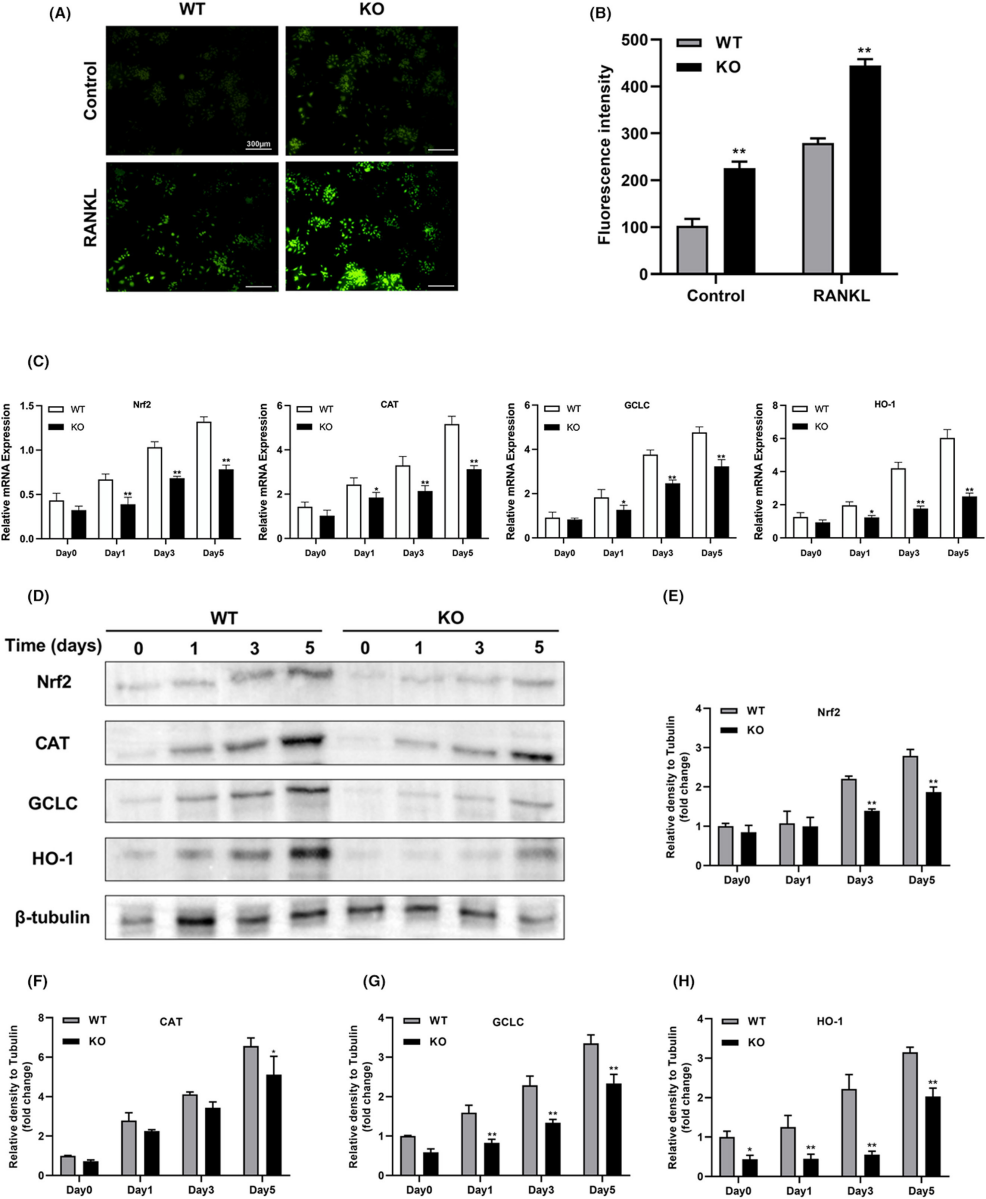
Deletion of MTA1 gene leads to increased ROS expression and decreased antioxidant enzyme expression during osteoclast differentiation. (A) Representative confocal images of RANKL‐induced ROS generation in RAW264.7 cells with WT and KO groups treated with RANKL (40 ng/mL), (Scale bar = 300 μm). (B) Quantification of relative DCF fluorescence intensity. (C) RAW264.7 cells in the WT and KO groups were stimulated with RANKL (40 ng/mL), and the expression of NRF2 and various antioxidant enzymes‐related RNA was detected by qRT‐PCR at 0, 1, 3 and 5 days of incubation. (D) RANKL (40 ng/mL) stimulated RAW264.7 cells WT and KO groups, and the expressions of NRF2 and several antioxidant enzymes were detected on incubation times (days 0, 1, 3 and 5) by western blot. (E‐H) Quantification of western blot bands for the protein levels of NRF2 and several antioxidant enzymes during osteoclast differentiation by ImageJ software. Data are expressed as the means ± SD of at least three independent experiments; *p < 0.05, **p < 0.01 versus WT group.

**FIGURE 7:**
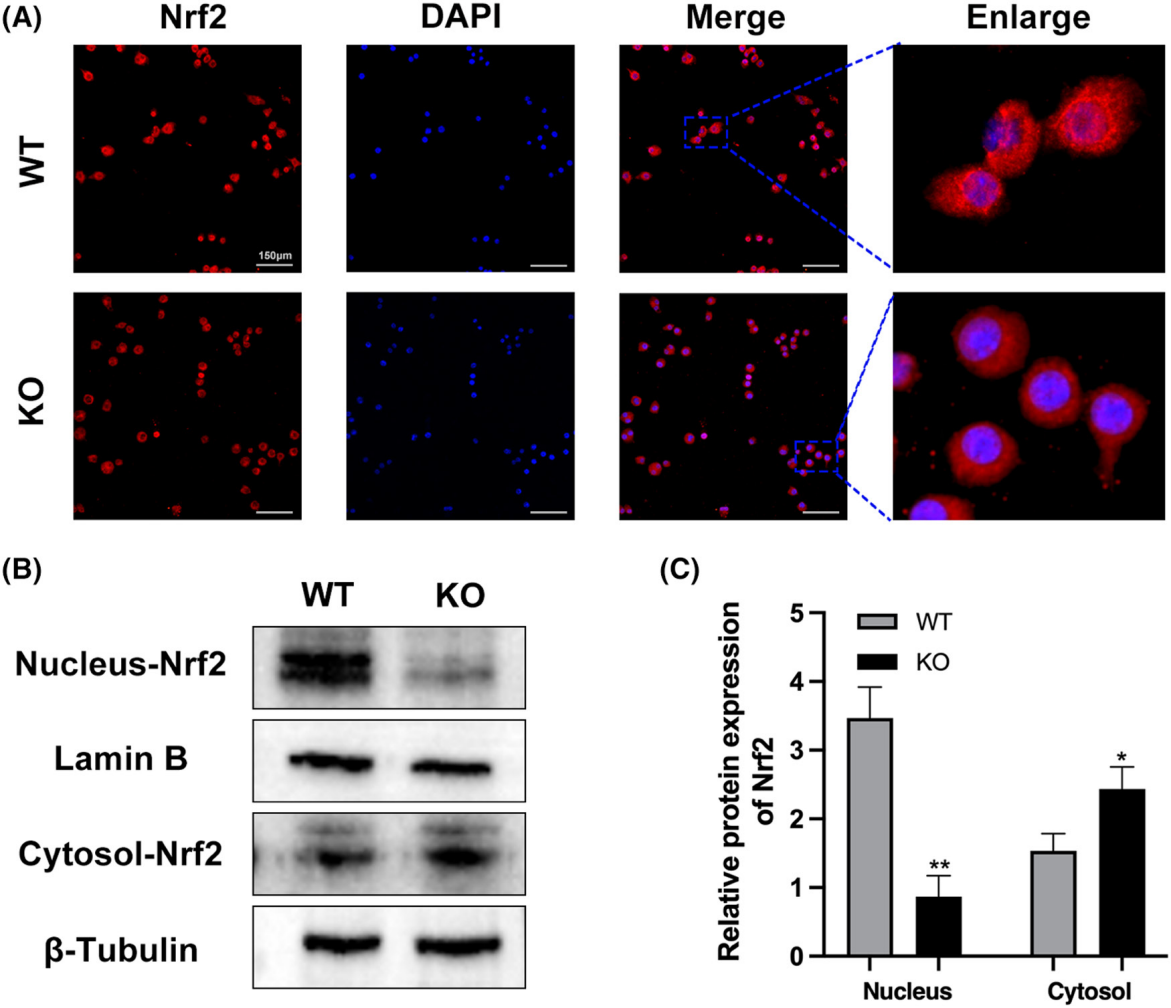
Effect of MTA1 knockdown on Nrf2 nuclear translocation induced by RANKL. (A) RAW264.7 cells were stimulated with 40 ng/mL RANKL for 24 h. Immunofluorescence staining (Scale bar = 150 μm) was performed to locate Nrf2 (red). Nuclear counter‐staining was performed using DAPI (blue). (B, C) RAW264.7 cells were stimulated with RANKL (40 ng/mL) for 24 h. Then, whole cytoplasmic and nuclear proteins were extracted and subjected to western blot analysis Nrf2. The levels of β‐tubulin and lamin B expression were measured to confirm equal protein amounts in cytoplasmic fractions and nuclear extracts. Data are expressed as the means ± SD of at least three independent experiments; *p < 0.05, **p < 0.01 versus WT group.

Under physiological conditions, ROS can regulate intracellular environmental homeostasis, signal transduction, proliferation and differentiation, which are in a dynamic balance with antioxidants.^19^ The inhibitory effect of ROS on osteoblasts and the promotion effect on osteoclasts will lead to greater bone resorption than bone formation, resulting in negative bone remodelling and ultimately reduced bone mass and bone strength. However, it is not clear whether MTA1 affects ROS production during osteoclastogenesis. We assessed control and RANKL‐induced intracellular ROS levels using DCFH‐DA staining. In the absence of RANKL stimulation, our results showed that ROS levels were higher in the KO group than in the WT group (Figure 6A, B). RANKL stimulation significantly increased ROS levels in both the WT and KO groups compared with the control group (Figure 6B). DCFH‐DA staining indicated that loss of MTA1 contributes to elevated intracellular ROS in both control and RANKL‐stimulated conditions. After oxidative stress and inflammatory reactions, activated NRF2 translocates to the nucleus and upregulates the expression of antioxidant enzymes such as heme oxygenase‐1 (HO‐1), catalase (CAT) and γ‐glutamyl cysteine synthetase catalytic subunit (GCLC).^20^ Total RNA was extracted at 0, 1, 3 and 5 days after RANKL induction. As shown in (Figure 6C), with osteoclast differentiation, antioxidant enzyme‐related genes (Nrf2, CAT, GCLC, HO‐1) showed a time‐dependent increase in both groups. However, the expression of antioxidase‐related genes in the KO group was significantly lower than that in the WT group. After that, proteins were extracted on day 0, 1, 3 and 5 to evaluate the protein expression of Nrf2 and its mediated antioxidant enzyme. As shown in (Figure 6D–H), the western blot results revealed that the Nrf2, HO‐1, CAT and GCLC levels were lower in the MTA1 KO group than the WT group during osteoclastogenesis. To further investigate whether MTA1 knockdown had an effect on nuclear translocation of NRF2, immunofluorescence was used to assess nuclear translocation of Nrf2, and western blot was used to assess Nrf2 protein levels in the nucleus and cytoplasm. As shown in Figure 7A, MTA1 knockdown significantly blocked nuclear translocation of Nrf2. In addition, western blot results also proved that the nuclear Nrf2 protein level was lower and the cytoplasmic Nrf2 level was higher in the KO group (Figure 7B, C). These collective data suggested that MTA1 deficiency enhances osteoclastogenesis via inhibiting nuclear translocation of Nrf2 and downregulating the expression of NRF2 and its induced antioxidants.

## 4 DISCUSSION

In the process of bone metabolism, under the synergistic action of relevant regulatory hormones and various cytokines, osteoblasts generate new bone and osteoclasts absorb old bone. This cycle repeats itself, eventually leaving the human bone homeostasis in a relatively stable state.^21^ Nevertheless, when bone resorption is increased or bone formation is reduced, the balance between bone resorption and bone formation is disrupted, leading to the destruction of the microenvironment and causing bone metabolism‐related diseases such as OP.^22^ The functional activation of osteoclasts is the key factor to induce osteoporosis pathology. At present, considering the limited of FDA‐approved drugs for osteolysis treatments and their side effects cannot be ignored.^23^ Consequently, the best treatment for OP should be to inhibit the differentiation of osteoclasts and promote the formation of osteoblasts without causing toxic and side effects. Active screening and identification of key molecules involved in the regulation of osteoclast differentiation and elucidation of their mechanisms will provide new therapeutic targets and intervention strategies for the treatment of OP. In this study, we elucidated for the first time the important role of MTA1 in osteoclast differentiation.

Metastasis‐associated protein 1 (MTA1), belonging to metastasis‐associated proteins (MTA) family, which are integral parts of nucleosome remodelling and histone deacetylation (NuRD) complexes. MTA1 is the earliest and most studied gene of MTA family. It regulates the expression level of tumour metastasis‐related proteins by regulating the transcription level and participating in signal transduction.^13^ Besides strong correlation between MTA1 upregulation and cancer, growing evidence strongly suggests that MTA1 could regulate divergent cell pathways by altering the status of key target genes in pathological and physiological states.^24^ It has been pointed out that MTA1 activated in an inflammatory response can coordinate with transglutaminase 2 to mediate inflammation, while MTA1 is also known to stimulate inflammation induced by sodium urate crystals.^25^ In addition, inactivation of MTA1 homologues egl‐27 and egr‐1 (also known as lin‐40) in *Caenorhabditis elegans*, leads to abnormal patterning of cells in the embryo, and forced overexpression of MTA1 in mouse mammary gland epithelium was accompanied by extensive ductal branching and proliferation in virgin glands, suggesting a role for MTA1 in both embryonic development and mammary gland development.^26^ A study by Liu et al. showed that MTA1 promoted osteoblast growth but inhibited the differentiation of MC3T3 cells under hypoxic conditions, suggesting that MTA1 plays an important role in bone metabolism.^16^ However, physiological characteristics of MTA1 have still been poorly addressed, especially the effects of MTA1 on osteoclast differentiation has not been reported so far. Considering that MTA protein plays a role in many physiological systems, especially in immune regulation, inflammation and cell differentiation, we speculated that the disorder of MTA1 expression may be directly involved in the pathogenesis of OP. Our study elucidated for the first time that MTA1 deficiency enhanced osteoclast formation in vitro. MTA1 may act as a negative regulator of RANKL‐induced osteoclast differentiation and maturation via affecting the activity of ROS pathway.

As has been demonstrated in previous studies, RANKL can independently induce RAW264.7 cells to differentiate into osteoclasts, and trap staining activity and nuclear number greater than 3 are generally defined as osteoclasts.^27^ TRAP staining results showed that compared with the control group, the RANKL induction group independently showed more TRAP‐positive multinucleated osteoclasts. In addition, osteoclasts are the only cells with bone resorption function in vivo, and their bone erosion function mainly depends on the formation of sealing zone on the bone surface by integrin protein and podosome. F‐actin staining results showed that the number of sealing zone in the RANKL group was significantly higher than in the control group. Meanwhile, western blotting and PCR results demonstrated that the expression of specific genes (c‐Fos, NFATc1, CTSK and MMP9) increased significantly with the increase of normal osteoclast differentiation. Interestingly, however, the expression of MTA1 protein and gene gradually decreased during osteoclast differentiation. Therefore, we speculated whether MTA1 affects the differentiation and function of osteoclasts. Subsequently, we used CRISPR/Cas9 gene editing technology to knockout MTA1 gene in the classical osteoclast model RAW264.7 cells. The results showed that with the differentiation of osteoclasts, MTA1‐deficient RAW264.7 cells formed more TRAP‐positive osteoclasts, and the formation of actin ring in mature osteoclasts was significantly increased. In addition, the expression of marker genes related to osteoclasts was significantly increased in the KO group. In summary, our results indicate that MTA1 may negatively regulates the differentiation, formation and function of osteoclasts.

In cells, ROS levels are in a dynamic and stable equilibrium. ROS are mainly derived from NADPH oxidase family (Nox) and mitochondrial respiratory electron transport chain (ETC) as by‐products of oxidative metabolic processes.^28^ Small amounts of ROS are used as native immune response or cell signalling molecules, and excess ROS causes oxidative stress by depleting glutathione and NADPH.^29^ Oxidative stress is an important factor in osteoporosis. Under the oxidative stress, ROS affects the expression of nuclear genes in osteoclast differentiation by activating a variety of cytokines and regulating multiple downstream signalling pathways.^30^ Besides, excessive ROS production damages the antioxidant system and promotes bone absorption and inhibits bone formation. Kelch‐like ECH‐associated protein1 (Keap1)/nuclear factor E2‐related factor 2 (Nrf2)/antioxidant response element (ARE) are one of the important endogenous antioxidant defence mechanisms in cells.^31^ In the physiological state, Nrf2 binds to Keap1 and becomes ubiquitinated in the cytoplasm, and when the binding site of Keap1 and Nrf2 becomes saturated, the newly synthesized Nrf2 translocates to the nucleus and induces the activation of a series of antioxidant enzymes (such as HO‐1, CAT and GCLC).^32^ When intracellular ROS levels ARE elevated, Keap1 dissociates from Nrf2, causing the latter to escape into the nucleus and bind to the ARE promoter region to promote the expression of antioxidant enzymes.^33^ Thus, Nrf2 maintains intracellular REDOX balance and protects cells and tissues from oxidative stress damage. However, it is not clear whether MTA1 regulates osteoclast differentiation by affecting oxidative stress. In this study, DCF fluorescence showed that intracellular ROS was significantly enhanced in the MTA1 knockdown group during RANKL‐induced osteoclast differentiation. Nrf2 and its transcription‐induced antioxidant enzymes were determined at the molecular level. The results showed that the mRNA and protein levels of Nrf2, HO‐1, CAT and GCLC increased in a time‐dependent manner during RANKL‐induced osteoclast differentiation, but were significantly lower in KO group than in WT group. Nuclear translocation of Nrf2 was assessed by immunofluorescence staining and western blot, and the results showed that MTA1 knockdown significantly inhibited Nrf2 nuclear translocation and more Nrf2 was retained in the cytosol. Our results suggested that MTA1 may regulate ROS and osteoclast differentiation and formation by influencing antioxidant production. Our study has some limitations. First, we demonstrated the role of MTA1 on RANKL‐induced osteoclast differentiation and maturation only in vitro. However, whether MTA1 affects the development of osteoporosis in preclinical animal models requires further investigation. Second, we only revealed the link between MTA1, Nrf2 and ROS during RANKL‐mediated osteoclast differentiation. Whether MTA1 plays an important role in osteoclast differentiation by regulating other signalling pathways remains to be established. Nonetheless, this study suggests a new hypothesis for the role of MTA1 in osteoporosis.

## 5 CONCLUSIONS

In this study, we demonstrate that MTA1 may negatively regulate osteoclast differentiation and formation by affecting ROS scavenging. MTA1 may be a potential target for the prevention and treatment of osteoporosis. As far as we know, this is the first study to investigate the role of MTA1 in osteoclast differentiation. More importantly, our study may open the way for MTA1 to become a new gene target for osteoporosis research.

## AUTHOR CONTRIBUTIONS

**Mingzhe Feng:** Data curation (equal); formal analysis (equal). **Lin Liu:** Data curation (equal); formal analysis (equal); validation (equal). **Zechao Qu:** Data curation (equal); investigation (equal); methodology (equal). **Bo Zhang:** Data curation (equal); formal analysis (equal); investigation (equal); validation (equal). **Yanjun Wang:** Conceptualization (equal); formal analysis (equal); resources (equal). **Liang Yan:** Investigation (equal); methodology (equal); visualization (equal). **Lingbo Kong:** Conceptualization (equal); resources (equal); software (equal); supervision (equal); writing – original draft (equal).

## FUNDING INFORMATION

This study was supported by a grant from National Natural Science Foundation of China (82070909).

## CONFLICT OF INTEREST STATEMENT

The authors declare that there are no conflicts of interest.

## Data Availability

All data and materials were included in the article.
